# Challenges and future options for the production of lutetium-177

**DOI:** 10.1007/s00259-021-05392-2

**Published:** 2021-05-11

**Authors:** W. V. Vogel, S. C. van der Marck, M. W. J. Versleijen

**Affiliations:** 1 Department of Nuclear Medicine, The Netherlands Cancer Institute-Antoni van Leeuwenhoek (NKI-AVL), Plesmanlaan 121, 1066 CX Amsterdam, the Netherlands; 2 Nuclear Research and consultancy Group (NRG), Petten, Netherlands

## Introduction

The use of the medical isotope lutetium-177 is increasing, but there are concerns that its worldwide availability may not be sufficient in the long term. This warrants an evaluation of its use and production.

Nuclear medicine involves the use of a spectrum of radioactive isotopes for diagnostic and therapeutic purposes, for example to identify cardiovascular and inflammatory diseases or to treat various types of cancer. The applied techniques require various isotopes with different physical properties. Isotopes for diagnostic purposes (gamma or positron emitters) typically decay with half-lives in the range of minutes to hours, while therapeutic isotopes (electron or alpha emitters) generally have half-lives of days to weeks. Because of this continuous loss by decay, in combination with the need to provide timely medical procedures for all patients in need, nuclear medicine requires uninterrupted and sufficient supply of relevant medical isotopes.

The applied medical isotopes have different requirements for production and distribution. For example, isotopes with longer half-lives like molybdenum-99 (^99^Mo) and lutetium-177 (^177^Lu) are typically produced by fission or activation in a nuclear reactor, and are then distributed to medical centres worldwide. Shorter lived isotopes cannot be transported over longer distances and need to be produced locally or regionally. Typical examples are technetium-99 m that is derived using a mother-daughter generator, and fluor-18 that is produced using a cyclotron. Continuous availability of all these production techniques at optimal locations is essential, in order to warrant adequate supply of every required isotopes throughout the world.

However, there is no central planning for the future of worldwide isotope production. In 2008–2010, the world started to notice this because the situation was dire: two of the main isotope-producing reactors at the time, the NRU in Canada and the HFR in the Netherlands, were both not operational unexpectedly, leading to a world-wide shortage of several medical isotopes. This caused important delays in diagnostic imaging and in treatment of cancer, and many centres struggled to find workable alternative strategies to provide adequate medical care for their patients [1]. This very challenging situation gave rise to international discussions, and efforts to improve the reliability and economic sustainability of the current production infrastructure world-wide. The OECD/NEA started the High Level Group Medical Radio-Isotopes (HLG-MR), and the EU started the European Observatory on the supply of medical radioisotopes. The situation also sparked new discussions about the future of reactor-based isotope production, and investments in nuclear production reactors are currently subject of political debate. In addition, several new alternative production methods have been proposed. Upon weighing the options for future production, it will be important to consider all relevant medical applications and their associated isotopes. However, recent discussions and proposed alternative techniques to generate isotopes are almost exclusively focussed at ^99^Mo [2]. Since production of different isotopes poses different technological challenges, this may lead to future situations where not all required isotopes can be produced with sufficient quantity, quality, reliability or geographic spread.

We identify ^177^Lu as an important example of a medical isotope that may be at risk for future shortages. The increasing demand for ^177^Lu is explained by its application in several current and new radionuclide therapies, most notably radiolabelled somatostatin analogs for neuroendocrine tumours and PSMA-ligands prostate cancer. In combination with the challenges related to production, this leads to concerns that the availability of ^177^Lu as a medical isotope may not be sufficient in the long term. In this editorial we discuss the distinctive clinical aspects of ^99^Mo and ^177^Lu, how their production routes are different, what the limitations of various available and proposed new production techniques are, and how limitations may compromise the future of various radionuclide therapies.

## Molybdenum-99

^99m^Tc is still considered the main workhorse of diagnostic nuclear medicine. Examples of routine applications include bone scintigraphy, functional renography, myocardial perfusion imaging, and the sentinel node procedure. ^99m^Tc has a relatively short half-life of 6 h and is therefore produced locally as a daughter nuclide using a ^99^Mo generator. As a result, sufficient supply of ^99^Mo is considered essential for diagnostic medical care.

The worldwide production of ^99^Mo is expressed in 6-day curie, which is the measured remaining radioactivity 6 days after it leaves the processor’s facility (measured from end of processing). After the supply problems in 2008–2010, the demand for ^99^Mo initially decreased from 12.000 to about 9.000 6-day Ci. But OECD studies show that this was followed by a renewed and ongoing upward trend, to 9.500 6-day Ci (351.500 GBq at 6 days after end of processing) ^99^Mo per week in 2019 [3]. The OECD expects that ^99^Mo demand will further increase annually by 0.5% in Europe and North America and by 5% in emerging markets. This outlook of slow demand growth was confirmed by NucAdvisor and Technopolis Group in a report for the European Union, based on among other things a survey among medical professionals [4]. It was concluded that the use of ^99m^Tc in Europe will remain stable until 2030. And even though it may decrease on the longer term, it is expected that SPECT(/CT) with ^99m^Tc will remain a major imaging technique.

The production of medical ^99^Mo is currently entirely reactor-based. After the prior shortages, the future of its production has been discussed intensively [3]. Around the same time, the non-proliferation effort to eliminate the use of high-enriched uranium gave a push towards changes in the conventional production chain. In recent years, several alternatives to conventional reactor-based production have been proposed, for example production of ^99^Mo using aqueous uranium solution reactors and direct production of ^99m^Tc using accelerators. Developments have shown that the most feasible route, involving the least risk to production levels, was to convert the existing reactor-based production routes to low-enriched uranium [5]. The challenges that had to be solved for the other production routes were too high: the radiochemical purity of the product, the massive scale-up of nuclear reactions with low probabilities of occurring (e.g., n + ^98^Mo → ^99^Mo), the compatibility with existing generators, or even the lack of regulations for new types of facilities. Ten years on, the result is that ^99^Mo is still produced in the conventional way, with most of the production now based on low-enriched uranium.

There are still a number of initiatives for isotope production facilities other than conventional reactors. Most of these started less than 10 years ago, as part of a drive for domestic ^99^Mo production in the USA [6]. In all these developments, that are discussed in more detail below, almost all attention still goes to ^99^Mo production alone.

## Lutetium-177

The medical isotope ^177^Lu is rapidly gaining ground as an additional workhorse in nuclear medicine. ^177^Lu decays to stable hafnium-177 with a half-life of 6.65 days, while emitting electrons up to 149 keV and with a maximum pathlength of 1.5 mm in tissue. ^177^Lu also emits photons up to 208 keV that are suitable for in-vivo biodistribution imaging using a gamma camera. Lutetium is suitable for radiolabelling of biologically active tracer molecules, with a similar chemical approach as the positron emitter gallium-68, to form a diagnostic/therapeutic pair. This allows treatment in conjunction with higher-resolution and quantitative diagnostic PET imaging for patient selection and response evaluation, a combination that is referred to as the theranostic approach [7]. These characteristics make ^177^Lu a very suitable isotope for image-guided radionuclide therapy [8].

The biodistribution of ^177^Lu was first reported in mice in 1978 [9], and this was followed by attempts to perform local radionuclide therapy with intra-articular injection in rats with arthritis [10]. After that, systemic administrations labelled to various experimental tracers were explored for multiple cancer types [11, 12]. The first mainstream application used ^177^Lu labelled somatostatin analogs to target well differentiated neuroendocrine tumours (NET) which usually overexpress the somatostatin receptor. This treatment is generally referred to as peptide receptor radionuclide therapy (PRRT) [13]. PRRT using ^177^Lu-DOTATATE is now a regular EMA/FDA-approved therapy, with proven efficacy for patients with metastatic or unresectable NETs [14]. ^177^Lu-DOTATATE therapy is currently also explored for several other tumour types, including for example melanoma, merkel cell carcinoma, esthesioneuroblastoma, thyroid cancer and malignant meningioma [15–19].

In the meantime, more biological targets have been identified that allow image-guided radionuclide therapy with ^177^Lu for other tumour types. Currently most promising is PSMA-directed therapy for patients with metastatic castration-resistant prostate cancer (mCRPC) who have no other effective treatment options left. In 2018 a meta-analysis of 10 studies with 455 patients receiving ^177^Lu-PSMA-617 therapy showed PSA response in 2/3 of patients already after one cycle, and this response was also associated with prolonged survival [20]. A recently completed multicenter phase 2 study with patients receiving up to 6 cycles Lu-PSMA at 6-week intervals (the TheraP trial), showed better PSA-response and lower toxicity as compared to cabazitaxel [21]. An international randomized phase 3 study is currently ongoing (the VISION trial), and its results are eagerly awaited [22]. A positive outcome of this study could facilitate registration of the applied ^177^Lu-PSMA-617, with the potential to become a new mainstream therapy for a large patient population. Other options currently under investigation include ^177^Lu labelled to ligands that target activated tumour-associated fibroblasts (FAPI) for various tumour types, and several other options [7, 23].

The current supply and demand situation for ^177^Lu is less transparent than for ^99^Mo. Since Lutathera currently is the only EMA/FDA-approved ^177^Lu-based medicine, it can be assumed that a sizable part of the ^177^Lu produced today is used in Lutathera. Based on the financial results published by Novartis and the list price of Lutathera, one would infer that the number of doses administered annually is around 10.000–15.000 doses of 7.4 GBq each. This is likely a strong underestimation of the total ^177^Lu production, if only because of significant research activity for the described new applications.

It is difficult to find an authoritative forecast of the ^177^Lu demand in open literature. Commercial market analysts expect a very significant growth in the demand for ^177^Lu in the coming years [7, 24], leading to the world demand for ^177^Lu multiplying several times over as a result of the introduction of new ^177^Lu-based medicines. The underlying reasons for this expected growth are easy to understand in global terms. The patient population that can be treated with ^177^Lu becomes much larger once PSMA-directed therapy becomes a regular treatment. Each year about 366.000 patients die of prostate cancer worldwide [25], of which a large part could theoretically benefit from PSMA-directed treatment with up to 6 cycles of around 7.4 GBq ^177^Lu. This could imply a worldwide potential for ^177^Lu-PSMA-ligand therapy of at least an order of magnitude more cycles (and total ^177^Lu dose) as compared to Lutathera. There are of course many assumptions going into this example, but it serves to illustrate the thinking behind the potentially spectacular growth that may be anticipated for ^177^Lu, even without considering other potential targets or patient groups that are currently under investigation.

## Production of Lutetium-177

**Fig. 1:**
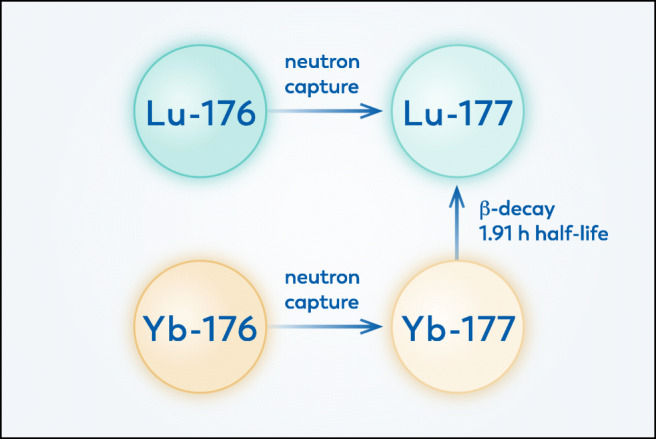
Illustration of possible production routes for ^177^Lu. Neutron irradiation of ^176^Lu yields carrier-added ^177^Lu, while neutron irradiation of ^176^Yb indirectly leads to no carrier added ^177^Lu via subsequent decay. There is no feasible production route possible without neutron irradiation

The increasing options and evidence for radionuclide therapy with ^177^Lu warrant an investigation of current and future production routes. ^177^Lu is produced in a nuclear reactor through irradiation of a source material with neutrons. There are currently two ways to produce ^177^Lu, the carrier added way and the no carrier added way, see Fig. 1 [26].

The carrier added route uses stable lutetium as the material to irradiate with neutrons. To enhance the yield, and to prevent the creation of unwanted other radionuclides, the lutetium is enriched in ^176^Lu. The first advantage of this route is that the nuclear reaction occurs with high probability, so that only one milligramme of enriched lutetium is needed to produce approximately 50 patient doses. The other advantage is that the radiochemical processing after irradiation is limited to producing lutetium chloride, which is relatively simple. This route also has some drawbacks. At the end of irradiation only about 30% of the lutetium is useful radioactive ^177^Lu. Since the ^177^Lu decays while the rest of the lutetium is stable, the fraction of useful lutetium is smaller by the time it reaches the patient. The other drawback of this route is that the irradiation not only produces ^177^Lu but also some ^177m^Lu, which is an unwanted beta emitter with a long half-life of 160 days that comes with additional challenges for example related to waste management.

The no carrier added route uses ytterbium as the material to irradiate with neutrons. As before, to enhance the yield, and to prevent the creation of unwanted other radionuclides, the ytterbium is enriched in ^176^Yb to a level above 99% [27]. The irradiation creates ^177^Yb, which has a half-life of under 2 h, decaying to ^177^Lu. One advantage of this route is that once the produced lutetium is separated from the ytterbium, the product consists of useful lutetium only with a high specific activity. The other advantage is that the decay of ^176^Yb to ^177^Lu does not co-produce the undesired ^177m^Lu. There are however also downsides to this route. Firstly, the nuclear reaction occurs with a low probability, so that a gram of enriched ytterbium is needed to produce the same amount of activity that is reached with only a milligramme of lutetium. Secondly, the lutetium needs to be separated chemically from the ytterbium, which is a challenging process: only 1 in roughly 5000 atoms in the irradiated material is a ^177^Lu atom, so the chemical separation process needs to have a high separation efficiency.

For both ways of producing ^177^Lu, a high neutron flux is needed, albeit for different reasons. For carrier added ^177^Lu, a low neutron flux would lead to a low specific activity (a low ratio of useful ^177^Lu as a fraction of all lutetium). This can be a restriction for possible success in therapy applications. For no carrier added ^177^Lu, a low neutron flux poses economic and chemical challenges. The required amount of enriched ytterbium is high from the start: for the same amount of ^177^Lu one requires a thousand times more enriched ytterbium than enriched lutetium. If the neutron flux is significantly lower than in current-days irradiation, the only way to compensate the lower yield is by increasing the mass of ytterbium that is irradiated. But enriched ytterbium is an extremely scarce product, making it excessively expensive. Currently, the only production of enriched ^176^Yb comes from low-throughput facilities (calutrons) in Russia [28], which is a problem anyway for the future of carrier-free ^177^Lu production. Lower neutron fluxes would not only aggravate this problem, but also increase the chemistry challenge: the reduced number of ^177^Lu atoms in the matrix of ytterbium below 1:5000 would require a separation efficiency that has, as yet, not been achieved.

It is not feasible to produce ^177^Lu by nuclear reactions that are initiated by something other than a neutron. There is no useful target that can be irradiated with an accelerated proton, or any other particle, to produce ^177^Lu. The only stable nuclides close to ^177^Lu on the nuclide chart apart from ^176^Yb and ^176^Lu are hafnium isotopes (e.g., ^178^Hf). Even the most promising nuclear reactions that would produce ^177^Lu via such routes are at least 50 times more unfavourable [29] than the reaction currently employed for no carrier added ^177^Lu, which itself is not very favourable. In addition, irradiation volumes in accelerators inherently need to be small. Therefore, accelerators cannot offer a viable alternative for large-scale ^177^Lu production.

## Types of production facilities

It follows that a high neutron flux is highly desirable, if not mandatory, for ^177^Lu production. Current production of ^177^Lu, both for carrier added and no carrier added ^177^Lu, is done in reactors with a high neutron flux (in alphabetical order): BR2 (Belgium), FRM-II (Germany), HFR (Netherlands), IVV-2 M (Russia), LVR-15 (Czech Republic), Maria (Poland), MURR (USA), OPAL (Australia), Safari (South Africa), and SM-3 (Russia). These facilities have been designed specifically to generate high neutron fluxes. Except for FRM-II and OPAL, these reactors are more than 40 years old. The question is whether new facilities with a low neutron flux, that have been designed almost exclusively for ^99^Mo production, can be competitive for ^177^Lu production. The answer to this question can be given by dividing the various proposed facilities in four categories.

### Photon beam facilities

There are two initiatives to produce ^99^Mo by irradiating ^100^Mo with a highly energetic photon beam: a photon with more than 10 MeV energy can knock a neutron out of the ^100^Mo nucleus, making it a ^99^Mo nucleus. NorthStar is nearing completion of a facility in Wisconsin (USA) [2]. This route also requires a new type of generator specifically designed to be compatible with the ^99^Mo that will be produced. The company IRE, in collaboration with ASML, plans to build a facility using the same nuclear reaction but with a significantly higher beam intensity [30]. This facility, SMART, is not as near to completion, but its ^99^Mo will be compatible with conventional generators. The NorthStar and Lighthouse/SMART facilities do not generate high neutron fluxes, and therefore they will not be competitive for ^177^Lu production.

### Accelerator-driven subcritical reactors

It is possible to use an accelerator to start the first neutrons, and subsequently use a so-called sub-critical assembly to multiply these neutrons [2]. This concept is used in the SHINE facility (accelerating deuterium atoms onto tritium atoms to create fusion, initiating neutrons) and in the Niowave facility (accelerating electrons onto a lead/bismuth target, leading to neutron production). These assemblies are in many ways similar to a nuclear reactor, with the important difference that the neutron chain reaction cannot sustain itself. As soon as the accelerator stops producing neutrons, the chain reaction stops.

There are limits associated with this method. One can only multiply the starting neutrons so many times. The higher the multiplication factor, the more this facility becomes a ‘normal’ reactor—which it is neither designed nor licenced to be. Another limit involves the cooling of the assembly. The design of these assemblies is not compatible with high-speed coolant flow, which acts as a limit on the neutron flux. Based on publicly available information on the size of the reactor cores and on the fission power produced in them, one can work out that the neutron flux is about 100× lower than in the reactors that currently produce ^177^Lu. Since a high flux is a primary requirement for ^177^Lu production, these facilities will not be competitive for ^177^Lu production.

### Power reactors

Bruce Power, Framatome and Kinectrics have a collaboration to start ^177^Lu production based on irradiations in Bruce Power reactors [31]. Contrary to all other reactors used for isotope production, these reactors are part of a nuclear power plant. The reactors are of the CANDU-type that allows on-line loading and unloading, which is necessary for producing isotopes with a half-life that is much shorter than the cycle length of a power plant. It is not immediately clear at which flux these irradiations take place, but it stands to reason to assume that the flux is high enough for a decent ^177^Lu yield. Also, the amount of space available in large reactors like these can be expected to be more than sufficient for this purpose. These reactors can be competitive for ^177^Lu production, provided one is willing to use the reactor space for this purpose, and willing to allow frequent loading and unloading of isotopes in a power station.

The catch with this initiative is that the isotope production clearly takes a back seat to electricity production. The latter brings in far more income, and when this becomes uneconomical for whatever reason, the isotope production will go down with it. In addition, it is important to note that the CANDU reactor type is the only one in which isotopes can be loaded/unloaded during operation, that only 7% of the power reactors is of this type, and that currently there is no CANDU reactor planned to be built or under construction. The newest CANDU reactor at the Bruce Power site in Canada started operations in 1987. In Europe there are only 2 operational CANDU reactors, both in Romania. It is questionable whether future isotope production should be dependent on the success and availability of CANDU reactors in the world of electricity production.

### Conventional ‘research’ reactors

There are a number of initiatives to build new reactors of the ‘research’ type. Perhaps a more appropriate name is ‘multi-purpose’ reactors, because often they can be used for isotope irradiations as well as for test irradiations of nuclear materials and fuels. For some of these initiatives, isotope production is one of the stated design goals, and in these cases a high flux is generally part of that design goal. As a result, these reactors will have a high neutron flux allowing them to be competitive for ^177^Lu production. Examples are KJRR (Korea), Pallas (Netherlands), and RA-10 (Argentina). One can also consider a number of initiatives for new reactors that may not have isotope production as their primary goal, but that could contribute to isotope production at some point in the future. Reactors such as JHR (France) and MYRRHA (Belgium) can be expected to be competitive for ^177^Lu production, at least if they will be engaged for this purpose.

A downside to the construction of new specifically designed reactors is the cost of development, that needs to generate a return on investment mainly through commercialization of the produced isotopes. This requires a long-term business case with regard to medical isotopes for diagnostic and therapeutic purposes.

## Discussion

In the coming years, production of medical isotopes will remain a matter of clinical, financial and political debate. There are multiple routes to production of ^99^Mo, potentially involving investments in several current and new techniques. But it remains a vital question whether future facilities, of which an increasing number may be optimized for ^99^Mo production alone, can also produce the full range of other required medical isotopes.

We identify ^177^Lu, which already is an indispensable isotope for radionuclide therapy and will become even more so with increasing number of treatable prostate cancer patients, as an important candidate isotope that may not be produced in sufficient quantities in the near future, in case of insufficient availability of high-flux neutron irradiation facilities. In 2015, Dash et al. provided a prior technical overview of production routes for ^177^Lu [26]. Since that time, new options for production routes were proposed and developed for ^99^Mo but not for ^177^Lu. We explained how the production of these isotopes is different, and why the production of ^177^Lu will require continued availability of high-flux neutron irradiation facilities.

There are many other nuclides with a potentially bright future in nuclear medicine, comparable to ^177^Lu, that have not been given sufficient attention even though they pose different requirements on the production technology. Examples of other therapeutic isotopes for which reactors with high neutron flux are essential include (but are not limited to) yttrium-90, samarium-153, iridium-192, tin-117 m, and lead-212. The reactors that produce ^177^Lu today are well equipped to produce these isotopes as well, but alternative technologies proposed for ^99^Mo production are ill suited for this purpose. As a result, the future of a broad range of isotopes depends on nuclear reactors of the conventional type.

The long-term supply of medical isotopes is becoming an urgent issue. While the isotope demand is set to increase over the coming years, current reactor facilities are generally quite old and their reliability for uninterrupted production will reduce over time. The construction of a new reactor facility requires political approval, funding, design, construction, and validation, a process that typically takes about 10 years in total. Political awareness of this challenge is crucial to ensure continued supply of all required medical isotopes. To achieve this, the ongoing debate about the relevance of nuclear production reactors needs to be supported by representatives of the medical field who have knowledge of the various production techniques and their limitations.

## Conclusions

We conclude that proposed new facilities for production of ^99^Mo alone will not be sufficient to maintain adequate supply of various important medical isotopes, with the highlighted example of ^177^Lu as an essential isotope for current and new radionuclide therapies for cancer. Continued investments in high-flux neutron irradiation facilities will be crucial to maintain sufficient availability of lutetium-177 and other medical isotopes, in order to provide required both diagnostic and therapeutic medical care for many patients in the coming years.
